# Multicenter retrospective analysis of clinicopathological features and prognosis of oral tongue squamous cell carcinoma in adolescent and young adult patients

**DOI:** 10.1097/MD.0000000000027560

**Published:** 2021-10-15

**Authors:** Kohei Okuyama, Souichi Yanamoto, Yasuyuki Michi, Eri Shibata, Maiko Tsuchiya, Misaki Yokokawa, Tomofumi Naruse, Hirofumi Tomioka, Takeshi Kuroshima, Hiroaki Shimamoto, Tohru Ikeda, Masahiro Umeda, Tetsuya Yoda, Hiroyuki Harada

**Affiliations:** a Department of Oral and Maxillofacial Surgery, Graduate School of Medical and Dental Sciences, Tokyo Medical and Dental University, Tokyo, Japan; b Department of Clinical Oral Oncology, Nagasaki University Graduate School of Biomedical Sciences, Nagasaki, Japan; c Department of Maxillofacial Surgery, Graduate School of Medical and Dental Sciences, Tokyo Medical and Dental University, Tokyo, Japan; d Department of Oral Pathology, Graduate School of Medical and Dental Sciences, Tokyo Medical and Dental University, Tokyo Japan; e Department of Dentistry and Oral surgery, Omura Municipal Hospital, Nagasaki, Japan.

**Keywords:** adolescents and young adults, disease-free survival, elective neck dissection, oral tongue squamous cell carcinoma, overall survival, therapeutic neck dissection

## Abstract

The aim of this study is to report the differences in clinicopathological features of oral tongue squamous cell carcinoma (OTSCC) and survival between adolescent and young adult (AYA) patients and elderly patients and to find the prognosticators. The medical records of 101 AYA patients and 175 control patients with OTSCC who underwent surgery were reviewed. Variables related to prognosis and their clinicopathological associations were analyzed. The 5-year overall survival (5y-OS) rates of AYA and control patients with stage I and II OTSCC were 94.4% and 89.6% (*P* = .353), respectively, and their 5-year disease-free survival (5y-DFS) rates were 82.0% and 76.6%, respectively (*P* = .476). The 5y-OS rates of patients with stages III and IV OTSCC were 83.3% and 66.7% (*P* = .333), respectively, and their 5y-DFS rates were 75.0% and 57.1% (*P* = .335), respectively. Logistic regression analysis revealed that there was no significant clinicopathological difference in AYA and control group. Furthermore, there was no significant difference in 5y-OS rates between patients who underwent elective neck dissection (END) and those who underwent therapeutic neck dissection (TND) in both group (*P* = 0.717 and 0.688). Overall, the present study revealed the clinicopathological features and prognosis of OTSCC were similar in AYA patients and elderly patients. Moreover, as there was no significant difference in OS between patients who underwent END and those who underwent TND in AYA and control groups, our results suggest that the indication for END in AYA patients with clinical N0 OTSCC is similar to that for elderly patients.

## 1 Introduction

Over the past few decades, treatments for cancers in both adult and pediatric patients have substantially improved, thus increasing the survival rates among these patient groups.^[1]^ Unfortunately, improvements in cancer treatment for adolescent and young adult (AYA) patients, specifically those between the ages of 15 and 39 years (the age range generally used to describe AYA patients in the United States), are lagging.^[2]^ In this generation, cancer remains a leading cause of death behind homicide, suicide, and injuries.^[3,4]^ The most common malignancies in AYA population are lymphoma, melanoma, testicular cancer, thyroid cancer, sarcoma, leukemia, central nervous system tumors, and breast cancer.^[2]^ AYA patients with these malignancies have not benefited from improvements in overall survival (OS), compared to adult or pediatric patients. One possible reason for this disparity may be that malignancies among AYA patients have unique biological characteristics, resulting in differences in clinical and treatment resistance behaviors.^[5]^ Moreover, managing cancer in AYA patients has several challenges owing to their unique clinical, psychological, and socioeconomic demands.^[6–8]^ In addition, the participation of AYA patients in clinical trials has been inadequate for many reasons, resulting in a relative lack of progress regarding advancements in treatments in this vulnerable population.^[9,10]^

Oral tongue squamous cell carcinoma (OTSCC) is a rare cancer and the most common histologic type of oral cancer, accounting for approximately 90% of cases.^[11]^ The differences in the clinicopathological features of early stage OTSCC between AYA and older adult patients were reviewed by Campbell et al.^[12]^ However, they did not report any relationship with survival. In the present study, considering that the first choice of treatment for OTSCC is surgical resection according to National Comprehensive Cancer Network guidelines,^[13]^ we assessed differences in clinicopathological features and survival between AYA and elderly patients in the context of OTSCC, with the aim of identifying specific prognostic factors associated with survival.

## 2 Materials and methods

### 2.1 Patients

According to the American Society of Clinical Oncology guidelines, AYAs were defined as individuals between the ages of 15 and 39 years.^[6]^ The authors retrospectively reviewed the medical records of 107 AYA patients who underwent surgery for oral squamous cell carcinoma (OSCC) between April 2008 and March 2017 at participating hospitals. The criteria for cervical lymph node metastasis (CLNM) were as follows: using computed tomography, magnetic resonance imaging, and/or neck ultrasonography, at least below were detected: the minor diameter of the lymph node over 10 mm, the intra-lymphatic heterogeneity, and/or a round shape of node. Therapeutic neck dissection (TND) was performed in patients who were clinically diagnosed with CLNM. Also, elective neck dissection (END) was performed in patients without CLNM who required simultaneous reconstruction for vascular anastomosis. Postoperatively, negative results were observed with a wait-and-see policy.

Since the most common age for OSCC development is the 60s,^[14]^ we excluded patients between the ages of the 40s and the 60s. Namely, the control group comprised patients in their 70s and 80s. This enabled us to clearly distinguish them from patients in the AYA group. A total of 420 patients with OSCC who met the aforementioned criteria were included in the control group. All eligible patients were capable of tolerating the surgical burden. After surgery, in patients with positive surgical margins, additional resection was performed. Postoperatively, all patients regularly underwent neck ultrasonography, computed tomography, and/or magnetic resonance imaging with or without contrast enhancement in their follow-up period. In the first year from the operation, patients visited the hospital at least once per month and underwent above imaging examinations every 3 to 6 months. In their second and third years, patients visited hospital at least once every 2 and 3 months, respectively. The follow-up period was then extended sequentially according to the duration from the operation.

### 2.2 Variables

The medical records, surgical procedures, clinicopathological findings, clinical courses, and prognoses were reviewed. The authors assessed age, sex, subsite of OSCC, disease stage (Union for International Cancer Control, version 7), and treatment outcomes, including local recurrence, occult CLNM (OCLNM), and distant metastasis. In addition, surgical specimens were assessed clinicopathologically for clinical type, tumor differentiation (World Health Organization grade), perineural invasion, lymphatic invasion, and vascular invasion. AYA and control groups were divided into the early (stages I and II) and late stage (stages III and IV) groups for analyses.

### 2.3 END and TND for AYA and control patients with OTSCC

A previous study reported that in OTSCC, one of the most important prognostic factors is the presence of neck metastasis.^[15]^ The survival of patients who underwent END versus TND in the AYA and control groups was assessed to determine the significance of END in AYA patients with clinical N0 OTSCC. The number of AYA patients who underwent END and TND was 21 and 13, respectively, and the numbers of control patients who underwent END and TND were 9 and 45, respectively. We analyzed the 5-year OS in patients who underwent END and TND to evaluate the validity of the “wait-and-see” policy in each group.

### 2.4 Statistical analysis

To find clinicopathological differences between the AYA and control groups, the associations between variables and groups were assessed using Fisher exact tests and multivariate logistic regression analysis. The associations between the events (local recurrence, OCLNM, and distant metastasis) and groups were analyzed using Fisher exact test. The 5-year OS and 5-year disease-free survival (DFS) rates in the early and late stages were compared using the log-rank test. OS was assessed from the date of diagnosis to the date of death or the last follow-up date for patients who were alive. DFS was assessed from the date of diagnosis to the date of recurrence, metastasis, death, or the last follow-up date. Statistical analyses were performed using SPSS version 25.0 (IBM Corp., Tokyo, Japan). Analysis items with two-tailed *P* values <.05 were considered statistically significant.

## 3 Results

### 3.1 The subsites of OSCC among AYA patients

The subsite of OSCC among almost all AYA patients was the tongue (101/107 patients, 94.4%), while the proportion of patients with OTSCC among control patients was only 41.7% (175/420 patients) (Table 1, bold value). Fisher exact test revealed a *P* value of <.001, indicating that the tongue is a significant OSCC subsite among AYA patients compared to that among control patients. Therefore, OTSCC patients were considered in all subsequent analyses. There was no significant bias in sex and stage among AYA and control patients.

**Table 1:** Clinical characteristics and clinicopathological features of patients in the present study.

	Patients	
Variable	AYA group (%) (n = 107)	Control group (%) (n = 420)
Age (yr)		
10s	1	–
20s	21	–
30s	85	–
70s	–	300
80s	–	120
Sex		
Female	50	203
Male	57	217
Subsite		
Tongue	101 (94.4)	175 (41.7)
Floor of the mouth	4	20
Buccal mucosa	1	52
Upper gingiva	0	64
Lower gingiva	1	97
Hard palate	0	6
Other	0	6
Stage		
I	61 (57.0)	180 (42.9)
II	32 (29.9)	128 (30.5)
III	8 (7.5)	33 (7.9)
IV	6 (5.6)	77 (18.3)
Unknown	0	2

### 3.2 Clinicopathological differences in OTSCC between the AYA and control groups

The OTSCC cohort in the AYA group included 47 women and 54 men with a median age of 33.0 (range: 21–39) years. The median follow-up period was 40.0 ± 30.6 (range: 1–132) months. In contrast, the control group included 70 females and 105 males with a median age of 76.0 years (range: 70–89). The median follow-up duration was 42.0 ± 28.8 (range: 1–130) months.

The clinical characteristics and clinicopathological features observed in the AYA and control groups during early- and late-stage OTSCC are summarized in Table 2. The differences observed in early stage OTSCC patients are as follows: the number of exophytic types in the AYA group tended to be lower, and perineural invasion, lymphatic invasion, and vascular invasion in the AYA group tended to be higher than in the control group. Univariate analysis revealed a significant difference in the clinical type of OTSCC in early stage. In contrast, multivariate analysis revealed that no variables were significantly associated with the AYA and control groups in both early and late stage. This result supports the previous study that compared the clinicopathology of early stage OTSCC between young and elderly adults.^[16]^

**Table 2:** Analysis of clinicopathological features in AYA and control patients with oral tongue squamous cell carcinoma.

		Stage I / II						Stage III / IV					
				Univariate analysis	Multivariate analysis					Univariate analysis	Multivariate analysis		
Variables		AYA group (n = 89)	Control group (n = 154)	*P* value	*P* value	OR	95% CI	AYA group (n = 12)	Control group (n = 21)	*P* value	*P* value	OR	95% CI
Clinical type	Superficial type	56	83	**.042**	.327	0.835	0.582 to 1.198	1	1	1.000	.922	0.926	0.199 to 4.310
	Exophytic type	8	32					2	3				
	Endophytic type	25	35					9	16				
	Unknown	0	4					0	1				
Tumor differentiation	Well	62	105	.903	.818	0.948	0.601 to 1.495	7	9	.722	.470	0.650	0.202 to 2.093
	Moderate	18	32					3	8				
	Poor	8	11					2	4				
	Unknown	1	6					0	0				
Perineural invasion	No	80	143	.331	.529	1.395	0.495 to 3.933	9	13	.703	.616	0.590	0.075 to 4.642
	Yes	9	10					3	8				
	Unknown	0	1					0	0				
Lymphatic invasion	No	77	140	.184	.362	1.551	0.604 to 3.983	7	14	.716	.555	1.682	0.299 to 9.451
	Yes	12	12					5	7				
	Unknown	0	2					0	0				
Vascular invasion	No	68	128	.170	.292	1.518	0.699 to 3.297	5	7	.716	.961	0.955	0.152 to 5.985
	Yes	21	24					7	14				
	Unknown	0	2					0	0				

### 3.3 Chemotherapy and radiotherapy

In the AYA group, adjuvant chemotherapy and radiotherapy were administered to 5 (4.9%) and 6 patients (5.9%), respectively, with multiple metastases with or without extranodal extension. In the control group, 10 patients (5.7%) received chemotherapy, and 14 patients (8.0%) underwent radiotherapy. According to the National Comprehensive Cancer Network guidelines,^[13]^ concurrent chemoradiotherapy with high-dose cisplatin is recommended for patients at high risk of recurrent and/or metastatic OTSCC, which includes patients with multiple CLNM, extranodal extensions, and positive surgical margins. In both groups, the adjuvant chemotherapy regimens used were mainly high-dose cisplatin which was combined with radiotherapy. In some cases, S-1 (tegafur–gimeracil–oteracil) was administered in combination with radiotherapy. This regimen was administered to patients with close surgical margins and renal disfunction and those who refused treatment with cisplatin. Total radiation doses ranged from 50 to 63 Gy. Radiotherapy at 12 Gy was stopped in 1 patient in the control group because of rapid growth of the tumor at the other side of the neck.

Patients with inoperable recurrence or metastasis were treated with chemotherapy or chemoradiotherapy. A regimen comprising cisplatin/5-fluorouracil with or without cetuximab was mainly administered to these patients as a first-line therapy. Treatment with one of the regimens was continued until response evaluation criteria in solid tumors-defined progression disease, unacceptable toxicity, or withdrawal of consent. The second-line therapy was cetuximab plus paclitaxel or maintenance with cetuximab alone. The completion rates for chemotherapy and radiotherapy were similar in both groups.

### 3.4 Survival in the AYA and control groups

**Figure 1:**
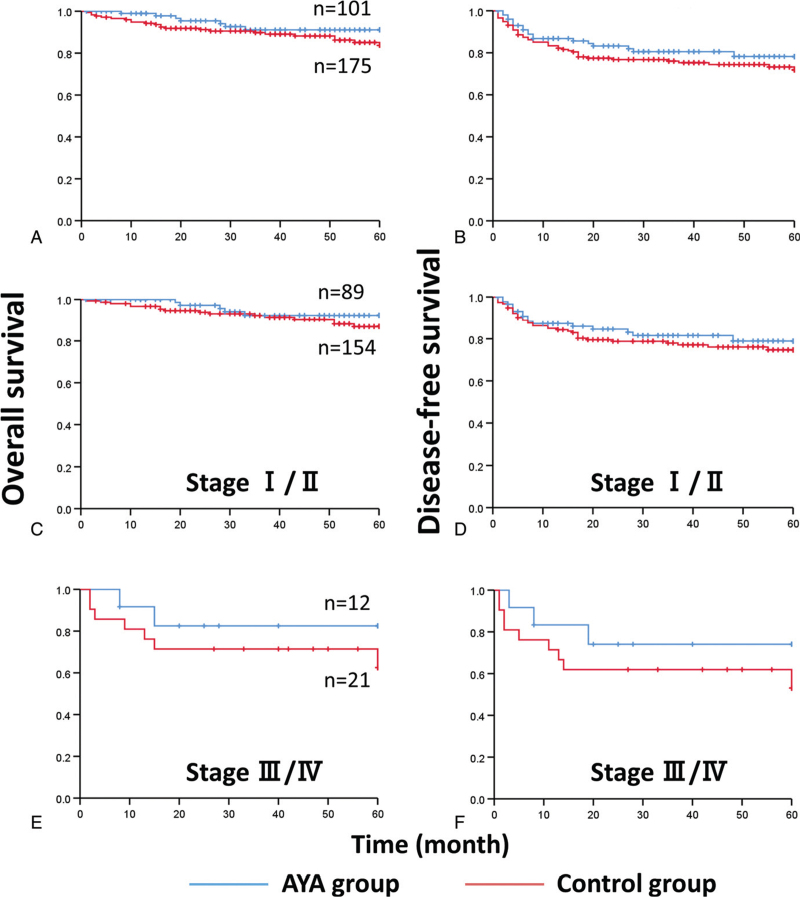
**(A)** The 5-year overall survival (5y-OS) rates of AYA and control patients in the present study were 93.1% and 86.9% (total 89.1%, *P* = .215), respectively, and **(B)** their 5-year disease-free survival (5y-DFS) rates were 81.2% and 74.3% (total 76.8%, *P* = .309), respectively. **(C)** The 5y-OS rates for patients with stages I and II OTSCC were 94.4 and 89.6% (total 91.4%, *P* = .353), respectively, and **(D)** their 5y-DFS rates were 82.2 and 76.6% (total 78.6%, *P* = .476), respectively. **(E)** The 5y-OS rates for patients with stages III and IV OTSCC were 83.3% and 66.7% (total 72.7%, *P* = .333), respectively, and **(F)** their 5y-DFS rates were 75.0% and 57.1% (total 63.6%, *P* = .335), respectively. AYA = adolescent and young adult, OTSCC = oral tongue squamous cell carcinoma.

There were no significant differences in stage-specific local recurrence, OCLNM, and distant metastasis between the 2 groups (Table 3). Figure 1 shows the Kaplan–Meier curves: the 5-year OS rates of AYA and control patients with stages I and II OTSCC were 94.4 and 89.6% (total 91.4%, *P* = .353), respectively, and their 5-year DFS rates were 82.2 and 76.6% (total 78.6%, *P* = .476), respectively. The 5-year OS rates of AYA and control patients with stages III and IV OTSCC were 83.3% and 66.7% (total 72.7%, *P* = .333), respectively, and their 5-year DFS rates were 75.0% and 57.1% (total 63.6%, *P* = .335), respectively. Although there was no significant difference, there was a trend toward poorer survival outcomes, both OS and DFS, in the control group, especially in the late stage.

**Table 3:** Postoperative courses among AYA and control patients with OTSCC.

	Patients n (%)		
Variable	AYA group n = 101	Control group n = 175	*P* value
Local recurrence			
No	95	167	.777
Yes	6 (5.9)	8 (4.6)	
Occult cervical lymph node metastasis			
No	80	141	.876
Yes	21 (20.8)	34 (19.4)	
Distant metastasis			
No	95	169	.366
Yes	6 (5.9)	6 (3.4)	

We also analyzed OS and DFS based on pathological results (p-stage). The 5-year OS rates of AYA and control patients with p-stage I and II were 98.8% (n = 84) and 89.9% (n = 149) (*P* = .022), respectively, and those of AYA and control patients with p-stage III and IV were 64.7% (n = 17) and 69.2% (n = 26) (*P* = .735), respectively. The 5-year DFS rates of AYA and control patients with p-stage I and II were 88.1% and 78.5% (*P* = .121), respectively, and the 5-year DFS rates for those with p-stage III and IV were 47.1% and 50.0% (*P* = .903), respectively.

### 3.5 END and TND in AYA patients with OTSCC

**Figure 2:**
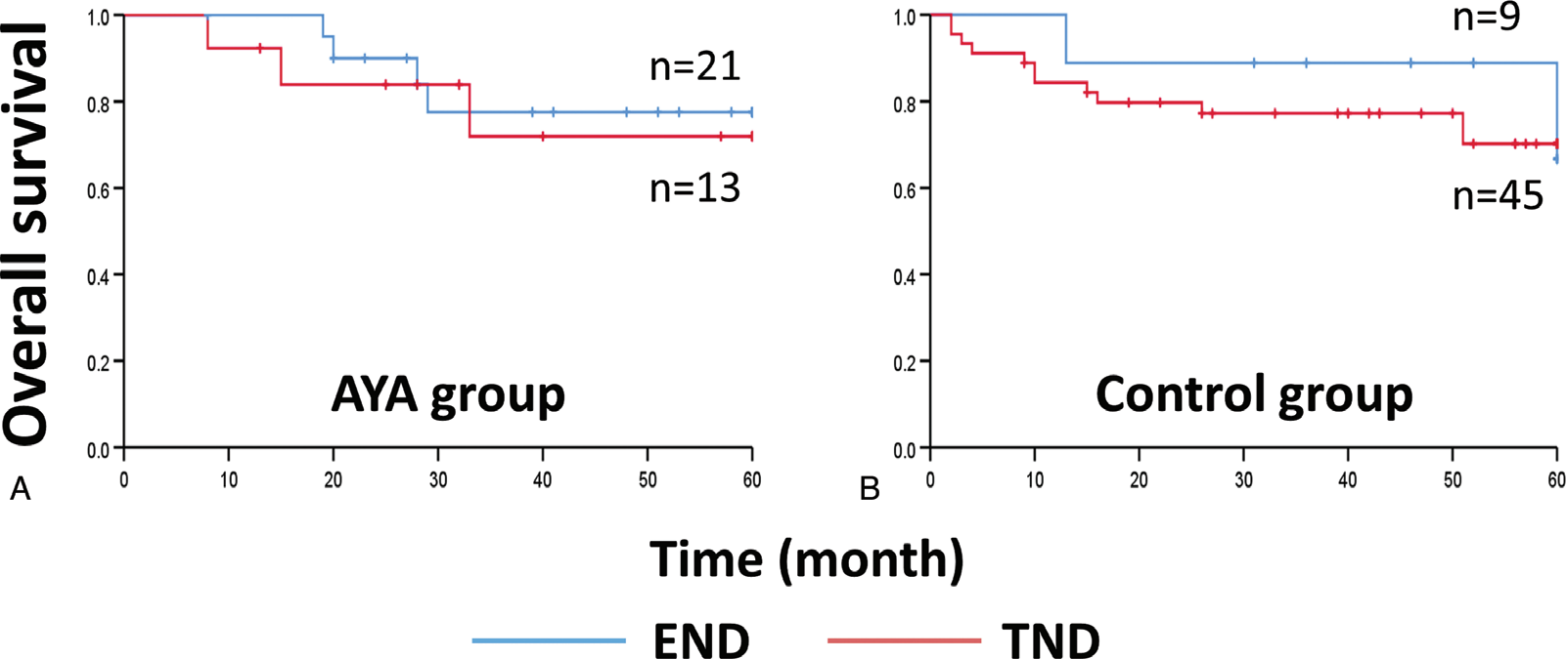
The 5-year overall survival rates of **(A)** AYA patients who underwent elective neck dissection and therapeutic neck dissection were 81.0% and 76.9% (total 79.4%, *P* = .717), respectively, and those of **(B)** control patients were 77.8% and 73.3% (total 74.1%, *P* = .688), respectively. AYA = adolescent and young adult, END = elective neck dissection, TND = therapeutic neck disection.

The 5-year OS rates of AYA patients who underwent END and TND were 81.0% and 76.9% (*P* = .717), respectively. In addition, the 5-year OS rates of control patients who underwent END and TND were 77.8% and 73.3% (*P* = .688), respectively (Fig. 2). Our findings suggest that the indication for END in AYA patients with clinical N0 OTSCC is similar to that for elderly patients.

## 4 Discussion

In the present study, we analyzed OTSCC patients whose ages are between 15 and 39 years according to the American Society of Clinical Oncology guidelines.^[6]^ However, this definition of the age has not been adopted worldwide.^[17]^ In the UK, teenage and young adult patients are considered to be those between 15 and 24 years of age; groups in New Zealand and Canada reference AYAs as individuals aged 15 to 29 years; and a publication from the Shanghai Cancer Institute on cancer incidence among AYAs included persons aged from 15 to 49 years.^[18–20]^ This discrepancy is considered as a variation of pediatric oncology practices in each country.^[21]^ Regardless of what is effectively an administrative or academic designation, these patients are represent patients with cancer who have unique needs.^[22,23]^

Cancers affecting AYA patients are diverse, spanning the spectrum from pediatric to adult-type malignancies. For instance, young women between the ages of 15 and 39 years are more likely to have high-grade, locally advanced triple-negative breast cancer than elderly patients,^[24]^ and young age appears to be a specific indicator of poor prognosis for this disease, independent of stage or histologic type.^[6]^ Thus, identifying and characterizing genomic mutations among cancers in AYAs may help us understand the role of disease biology in determining prognosis and predicting therapeutic outcomes. In head and neck oncology, Ryu et al^[25]^ reported that perineural invasion, PD-L1 positivity, and a higher ratio of CD163-positive tumor infiltrating macrophages to CD8-positive tumor infiltrating lymphocytes were independent factors for poor progression-free survival in young patients. However, the ability to apply such findings to routine clinical practice is limited by high costs, special techniques, and equipment associated with sequencing, and the inability to validate these findings in all hospitals.

Notably, almost all OSCCs occurred on the tongue in patients with AYA in the present study. The general causes of OTSCC have been reported to include unsuitable tooth fillings or prosthesis placement, smoking, alcohol consumption, chronic inflammation, precancerous lesions such as epithelial dysplasia, infection, endocrine disease, poor oral hygiene, heredity, mechanical trauma, galvanic phenomena, and contact allergy to metal dental restorations.^[12,26–32]^ However, since the duration of the exposure to the aforementioned causes is apparently shorter in AYA patients than in elderly patients, those causes cannot be applied for the development of OTSCC in AYA patients. Kim et al^[33]^ reported that the lingual position of the mandibular second molar and the narrow tongue space in young mature patients are associated with tongue cancer development. Therefore, we believe that AYA patients with OTSCC tend to have specific anatomical physical characteristics. The association between these features and the development of OTSCC among AYA patients should be prospectively investigated in the future. In the AYA population, Fanconi anemia is also a strong risk factor for the development of head and neck SCC because of the absence of DNA repair genes.^[34,35]^ The present cohort also included 3 AYA patients with Fanconi anemia.

In the present study, the 5-year OS and DFS rates were not significantly different between the AYA and control groups, the recurrence and metastasis rates were similar in both groups, and there was no significant difference in the completion rates of chemotherapy and radiotherapy between the 2 groups. Taken together, considering that there were no significant clinicopathological and survival differences in both groups, OTSCC among AYA patients is not always characterized by increased aggressiveness compared with that in elderly patients. In contrast, Friedlander et al^[36]^ previously reported that younger patients with OTSCC had a significantly higher rate of locoregional recurrence than older patients; however, the 5-year DFS rates in the young and older groups were not significantly different. Verschuur et al^[37]^ also reported that young patients with head and neck SCC did not have a worse prognosis than a matched older patient group in their case-controlled study. Our data also support these results and the results of a previous meta-analysis by Pitman et al.^[38]^ Furthermore, Oliver et al^[39]^ reported that their propensity score-matched survival analysis in the National Cancer Database revealed that OTSCC patients aged under 40 years had a 9% higher 5-year survival rate. They concluded that younger patients with OTSCC did not have worse survival (77.1% vs 68.2%, *P* < .001).^[39]^

In the present study, the rate of OCLNM was similar between the 2 groups (20.8% and 19.4%). In addition, there was no significance in the 5-year OS rates of AYA and control patients who underwent END and TND, suggests that the indication for END for AYA patients is similar to that for elderly patients. In a systematic review and meta-analysis, Abu-Ghanem et al^[40]^ reported that END can significantly reduce the rate of regional nodal recurrence and improve disease-specific survival rate but cannot improve OS in patients with clinical T1/2 N0 OTSCC. They reported that the “wait-and-see” policy did not decrease OS in patients with early-stage OTSCC. In contrast, D’Cruz et al^[41]^ reported in a prospective, randomized, controlled trial that among patients with early stage OSCC, END resulted in higher OS and DFS than TND. Although they insisted on the significance of END, their study design is far different from ours. Their cohort included patients with OSCCs other than OTSCC. Their follow-up interval and the timing of their routine imaging examination were also different from ours. Therefore, their END criteria may not directly apply to the AYA cohort in our study. In addition, tumor depth is also a controversial potential factor. Otsuru et al^[42]^ reported that END should be performed in OTSCC patients with a tumor depth of at least 4 to 5 mm, which is associated with a high rate of OCLNM. Considering that the univariate analysis in the present study revealed that the clinical type of OTSCC in the early stage is a significant difference between the AYA and control OTSCC patients, prospective studies involving large numbers of AYA patients will help to determine the efficacy of this parameter.

The present study had some limitations, mostly pertaining to its retrospective design, including cohort selection, the impact of previous exposure to risk variables (eg, smoking, alcohol consumption, and virus status), treatment approaches, follow-up, reporting (including missing data), complications (eg, diabetes mellitus and immunosuppressive conditions), and genetic mutations (eg, Fanconi anemia).

In conclusion, there were no significant differences in clinicopathological features and survival between the AYA and control group. Moreover, since there was no significant difference in OS between patients who underwent END and those who underwent TND in the AYA and control groups, our results suggest that the indication for END for AYA patients with clinical N0 OTSCC is similar to that for elderly patients.

## Author contributions

**Conceptualization:** Kohei Okuyama.

**Data curation:** Kohei Okuyama, Souichi Yanamoto, Yasuyuki Michi, Eri Shibata, Maiko Tsuchiya, Misaki Yokokawa, Tomofumi Naruse, Hirofumi Tomioka, Takeshi Kuroshima, Hiroaki Shimamoto, Hiroyuki Harada.

**Formal analysis:** Kohei Okuyama, Souichi Yanamoto.

**Investigation:** Kohei Okuyama, Souichi Yanamoto, Yasuyuki Michi.

**Project administration:** Kohei Okuyama.

**Software:** Kohei Okuyama.

**Supervision:** Souichi Yanamoto, Tohru Ikeda, Masahiro Umeda, Tetsuya Yoda, Hiroyuki Harada.

**Validation:** Kohei Okuyama, Masahiro Umeda, Tetsuya Yoda, Hiroyuki Harada.

**Writing – original draft:** Kohei Okuyama.

**Writing – review & editing:** Kohei Okuyama.
